# Independent Relevance of Estrogen Receptor and Progesterone Receptor Statuses in DCIS on Risk of Subsequent Ipsilateral and Contralateral Invasive Breast Events in Absence of Endocrine Therapy

**DOI:** 10.3390/cancers18071109

**Published:** 2026-03-30

**Authors:** Thomas J. O’Keefe, Audrey Guo, David R. Vera, Anne M. Wallace

**Affiliations:** 1 Department of Surgery, University of California San Diego, San Diego, CA 92093, USA; 2 Torrey Pines High School, San Diego, CA 92130, USA; 3 Department of Radiology, University of California San Diego, San Diego, CA 92093, USA

**Keywords:** DCIS, biomarkers, breast cancer, cancer outcomes, estrogen receptor, progesterone receptor, risk stratification

## Abstract

Background: Patients with estrogen receptor (ER)-positive ductal carcinoma in situ (DCIS) derive a greater benefit from endocrine therapy than patients with ER-negative disease. The relevance of ER status and progesterone receptor (PR) status in DCIS to radiation therapy has not been well explored. Methods: Patients undergoing breast-conserving surgery with or without radiation were grouped by ER and PR status and matched using rank-based Mahalanobis optimal matching with respect to lesion size and grade and patient age and race. Cumulative incidences were estimated and competing risk regressions with subdistribution hazard ratios (sHRs) were calculated. Results: Among patients who underwent breast-conserving surgery only, 369 patients with ER-PR- disease were matched to 738 patients with ER+PR+ disease (1:2 matching). In multivariate models, patients with ER-PR- disease were at increased risk of any invasive events (sHR = 2.47, *p* = 0.007) and early ipsilateral invasive events (sHR = 2.64, *p* = 0.02 in the 0-to-4-year period) relative to patients with ER+PR+ disease. Among patients who underwent breast-conserving surgery with adjuvant radiation, 1498 patients with ER+PR+ disease were matched to 1498 patients with ER-PR- disease. No significant differences were noted with respect to cumulative incidence of any invasive event (5.6% vs. 5.6%) or ipsilateral invasive events (1.9% vs. 2.9%). In multivariate models, no significant differences were noted. Patients with ER-PR+ lesions had similar cumulative incidences of ipsilateral invasive events to patients with ER-PR- disease in the absence of radiation (5.9% vs. 5.9%) and similar cumulative incidences of contralateral invasive events to patients with ER+PR+ disease when radiation was administered (3.2% vs. 4.2%). Conclusion: The statuses of ER and PR carry independent prognostic and therapeutic implications beyond those of traditional clinicopathologic risk factors. Given that ER and PR statuses are routinely collected for patients with DCIS, incorporation of these variables into clinicopathologic risk classification systems is warranted.

## Simple Summary

Patients with ER-PR- DCIS treated with breast-conserving surgery without radiation are at increased risk of subsequent invasive events relative to patients with ER+PR+ DCIS. When treated with breast-conserving surgery and external beam radiation, patients with ER-PR- and ER+PR+ disease had similar rates of ipsilateral and contralateral subsequent invasive events. Patients with ER+PR- disease had similar ipsilateral risk to patients with ER-PR- lesions and similar contralateral risk to patients with ER+PR+ lesions when accounting for treatment. These results suggest that radiation may provide the greatest benefit from an invasive recurrence standpoint for patients with ER-PR- DCIS.

## 1. Introduction

Patients with ductal carcinoma in situ (DCIS) are at increased risk for the development of subsequent ipsilateral invasive disease [1,2]. Screening mammography resulted in a steep increase in the incidence of DCIS [3]. A series of randomized trials were conducted to assess whether patients with lesions amenable to breast-conserving surgery would benefit from adjuvant radiation therapy. All of these trials found that radiotherapy reduced ipsilateral in situ and invasive subsequent events [4,5,6,7,8]. Two trials assessed the effect of five years of tamoxifen, with conflicting results: NSABP B24 found that the addition of tamoxifen to radiotherapy reduced ipsilateral and contralateral invasive events, while the UK/ANZ trial found that endocrine therapy had no effect on patients who received radiation [8,9]. Subsequent retrospective analyses of these two trials found that patients with estrogen receptor (ER)-positive lesions were at lower risk for ipsilateral subsequent events than patients with ER-negative lesions, and also responded better to tamoxifen [10,11]. Importantly, while these trials found that radiation and endocrine therapy reduced invasive subsequent events, and patients who had invasive subsequent events were at significantly elevated risk of ultimate breast cancer mortality, none of these trials found a breast cancer-specific or overall survival benefit with radiation or endocrine therapy [5,6,8,9,12]. It is unlikely that this is secondary to sample size, as a meta-analysis of the radiotherapy trials found that radiation was associated with a non-significantly increased risk of breast cancer mortality [4].

The current standard of care from the National Comprehensive Cancer Network (NCCN) guidelines for DCIS includes breast-conserving surgery, adjuvant radiation therapy, and consideration of 5 years of endocrine therapy for patients with estrogen receptor (ER)-positive lesions [13]. However, given that the majority of women who undergo breast-conserving surgery without any adjuvant treatment will not have an ipsilateral subsequent event [5,9,14], and even fewer will have an invasive subsequent ipsilateral event [5,6,9], this standard represents overtreatment for the majority of patients. Traditional clinicopathologic risk scoring systems have shown relatively poor performance for the stratification of risk for DCIS patients [12,14,15,16]. More recently, increasing attention has been paid to molecular tests for risk stratification. The two best-known of these tests are Oncotype DX DCIS [17] and DCISionRT [18]. Oncotype DX DCIS has some predictive value, but in adjusted models it is less predictive than other standard clinicopathologic risk factors [19,20]. DCISionRT has gained popularity in recent years and received FDA Breakthrough Device Designation in 2025. The test has demonstrated some success in retrospective analyses, but questionable practices such as a validation of the test’s superiority over clinicopathologic risk scoring systems using a dataset from which 36% of the cohort had previously been used to train the DCISionRT model [21] casts some doubt on these reports. Furthermore, the only report on the test’s efficacy using prospective, randomized data was far less impressive than its performance in retrospective cohorts [22].

While both Oncotype DX DCIS and DCISionRT incorporate progesterone receptor (PR) status into scoring, neither of them incorporates ER status. The report on the development for Oncotype DX DCIS attributed this to its primary relation being with respect to endocrine therapy responsiveness [17]. However, this appears to have been largely assumed from invasive cancer, as over 95% of patients used in the development of the test had invasive rather than in situ cancer [17], and ER has been shown to have not only therapeutic but also prognostic relevance in DCIS [11]. This may also relate to the fact that most PR+ DCIS is driven by ER+ status, making ER-PR+ DCIS quite rare. Despite being assessed as part of the standard of care [13], ER status is not included in clinicopathologic risk scoring systems. Here, we aim to assess whether ER and PR statuses hold independent prognostic or therapeutic significance among patients who do not receive endocrine therapy by comparing outcomes to patients with lesions matched on traditional clinicopathologic risk factors in the context of treatment with breast-conserving surgery only or breast-conserving surgery with adjuvant radiation.

## 2. Methods

### 2.1. Ethical Considerations

The UCSD Institutional Review Board deferred approval of this study due to its use of publicly available and deidentified national data. The study was conducted in accordance with US Common Rule.

### 2.2. Patient Selection

We used data from the Surveillance, Epidemiology and End Results (SEER) 17 registry (November 2023 submission). Women with a first cancer of unilateral DCIS diagnosed at age 75 years or younger with at least 6 months of follow-up and known cause of death, year of diagnosis, patient race, lesion laterality, lesion grade, surgery type, ER status, and PR status were included. Only patients who underwent breast-conserving surgery with or without beam radiation were included. We excluded patients who underwent mastectomy both because of the anticipated very low rate of ipsilateral invasive subsequent events and because of problems with respect to how breast cancer mortality is coded for such patients [23]. Patients were excluded if they had known HER2 status because HER2 testing is not standard of care for patients with DCIS and may influence the risk of ipsilateral invasive recurrence [24,25]. Other exclusion criteria included multicentric disease and lesion size greater than 3 cm. The size cutoff was selected because (i) large lesions can require the use of non-standard extreme oncoplasty techniques which may introduce bias [26,27], (ii) very large DCIS has been associated with increased risk of breast cancer mortality [28], and (iii) SEER does not report surgical margins and larger lesion size has been associated with increased risk of positive surgical margins [29], which in turn is associated with risk of ipsilateral invasive recurrence in patients who do not receive both radiation therapy and endocrine therapy [9]. The age cutoff was selected to mitigate the impact of the competing risk of non-breast cancer mortality for older women. Patients were only included if they were diagnosed at least eight years before the year of final follow-up for data in the SEER iteration that was used to allow for comparable maximum possible follow-up. Patients who received chemotherapy or neoadjuvant radiation therapy were excluded because this is not recommended for patients with pure DCIS. Patients who received endocrine therapy were excluded as it has known therapeutic and prognostic relevance and would confound the results [9,30], and because the aim of the study was to assess the prognostic and therapeutic significance of these factors outside of endocrine therapy relevance. Patients who underwent a nipple-sparing mastectomy were excluded as the laterality of this surgery is not coded in SEER. Ipsilateral and contralateral invasive subsequent diagnoses and times to these diagnoses were gathered, and a composite variable of “any invasive event” was derived from them to represent the first of any invasive subsequent events that the patient had, independent of laterality, as well as breast cancer mortality in the absence of an in-breast invasive event.

### 2.3. Analysis

Four matched cohorts were assembled. For the first two cohorts, matching was performed with respect to the variable hormone receptor status, which was defined as positive if ER and PR were both positive and negative if ER and PR were both negative. The first cohort involved matching patients who underwent breast-conserving surgery without radiation. The second involved matching patients who underwent breast-conserving surgery with adjuvant beam radiation therapy. The third cohort involved matching patients with ER+PR- disease who underwent breast-conserving surgery without radiation to patients with ER-PR- disease and separately to patients with ER+PR+ disease. The fourth cohort involved matching patients with ER+PR- disease who underwent breast-conserving surgery with adjuvant radiation to patients with ER-PR- disease and separately to patients with ER+PR+ disease.

Optimal rank-based Mahalanobis pair matching was performed with tumor grade, size, age, and race, with separate exact matching for tumor grade (categorized as low or intermediate vs. high or undifferentiated). Furthermore, matched patients were required to have lesion sizes within 5 mm of one another and ages within 5 years of one another. Matching was performed 2:1 for the first cohort and 1:1 for the second cohort, as the latter could not be matched appropriately in a 2:1 fashion for the specified matching parameters. For the third and fourth cohorts, matching was performed 1:1 for ER+PR- disease to ER-PR- disease and 1:2 for ER+PR- disease to ER+PR+ disease, again because this was possible in the former but not the latter due to relatively larger numbers of patients with ER+PR+ disease. Cumulative incidences of invasive events and breast cancer mortality were estimated using Fine and Gray competing risks methods [31].

Non-breast cancer death was considered as a competing risk for subsequent invasive diagnoses. Ipsilateral and contralateral invasive diagnoses were analyzed separately as well, and death from any cause was treated as the only competing event for both of these. Multivariate models were adjusted for patient age, tumor size, tumor grade, race and treatment, and subdistribution hazard ratios (sHRs) were calculated. Treatment was incorporated as a time-dependent coefficient using a step function with a cutoff at 4 years, selected to divide the 8-year follow-up period in half [32]. Statistical significance was declared for *p* less than 0.05. All statistical analyses were performed in R (4.4.1, R Foundation for Statistical Computing, Vienna, Austria) using RStudio (2024.09.0+375) and the packages “optmatch” (0.10.8), “tidyverse” (2.0.0), “cmprsk” (2.2–12), and “survival” (3.8–3) [33,34,35].

## 3. Results

### 3.1. Unmatched Breast-Conserving Surgery Without Radiation Therapy Cohort

In total, 3550 patients met inclusion criteria for the first cohort (ER+PR+ or ER-PR- undergoing breast-conserving surgery without radiation) and were eligible for matching, among whom 3181 (89.6%) had ER+PR+ disease and 369 (10.4%) had ER-PR- disease. Median age was 58 years (interquartile range [IQR] 50–66) and median lesion size was 7 mm (IQR 4–12). In total, 2775 (78.2%) were white, 309 (8.7%) were Black, and 466 (13.1%) were of a non-white, non-Black race. Overall, 759 (21.4%) of patients had low-grade disease, 1751 (49.3%) had intermediate-grade disease, 820 (23.1%) had high-grade disease, and 220 (6.2%) had undifferentiated-grade disease. During the 8-year follow-up period, 134 (3.8%) patients had an invasive ipsilateral event, 83 (2.3%) had a contralateral invasive event, 26 (0.7%) died of breast cancer, and 191 (5.4%) died of causes other than breast cancer. Within this unmatched cohort, the 8-year cumulative incidence for patients with ER+PR+ relative to ER-PR- lesions was 6.1% vs. 9.6% for any invasive event, 3.6% vs. 6.0% for ipsilateral invasive events, 2.3% vs. 3.3% for contralateral invasive events, and 0.6% vs. 1.6% for breast cancer mortality.

### 3.2. Matched Breast-Conserving Surgery Without Radiation Therapy Cohort

A total of 1107 patients were matched at a 2:1 ratio including 369 patients (33.3%) with ER-PR- disease and 738 patients (66.7%) with ER+PR+ disease (Table 1). Median age was 59 years (IQR 52–67). Median size was 10 mm (IQR 5–17). In total, 869 (78.5%) of patients were white, 97 (8.8%) of patients were Black, and 141 (12.7%) of patients were of a non-white, non-Black race. Overall, 21 (0.5%) patients had low-grade disease, 207 (18.7%) had intermediate-grade disease, 684 (61.8%) had high-grade disease, and 195 (17.6%) had undifferentiated-grade disease. During the 8-year follow-up period, 45 (4.1%) patients had an ipsilateral invasive event, 28 (2.5%) had a contralateral invasive event, 11 (1.0%) patients died of breast cancer, and 57 (5.1%) patients died of causes other than breast cancer.

**Figure 1:**
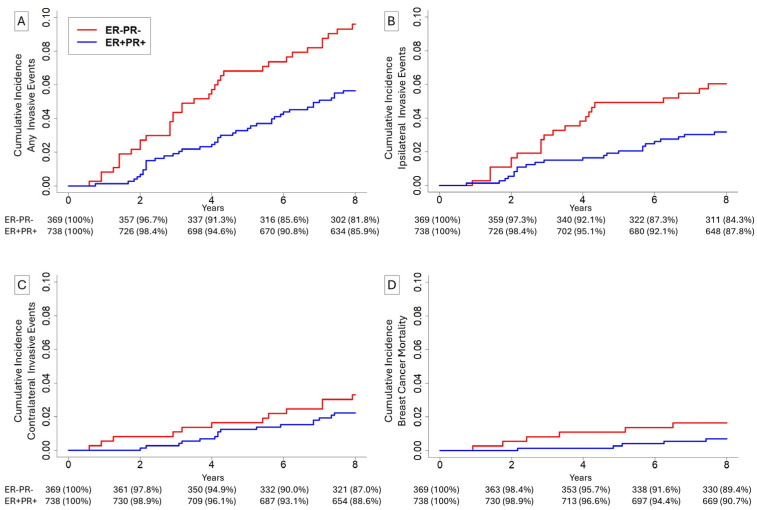
Cumulative incidence of (**A**) any invasive events, (**B**) ipsilateral invasive events, (**C**) contralateral invasive events, and (**D**) breast cancer mortality for patients undergoing breast-conserving surgery without radiation with estrogen-receptor-positive (ER+) and progesterone-receptor-positive (PR+) DCIS, or ER-PR- DCIS.

Within this matched cohort, the 8-year cumulative incidence for patients with ER+PR+ relative to ER-PR- lesions was 5.7% vs. 9.6% (Figure 1A, *p* = 0.01) for any invasive event, 3.2% vs. 6.0% (Figure 1B, *p* = 0.02) for ipsilateral invasive events, 2.2% vs. 3.3% (Figure 1C, *p* = 0.28) for contralateral invasive events, and 0.7% vs. 1.6% (Figure 1D, *p* = 0.13) for breast cancer mortality. In adjusted multivariate models (Table 2), ER-PR- disease was associated with increased risk of any invasive subsequent event in the 0-to-4-year time frame (sHR = 2.47, *p* = 0.007) and unchanged risk in the 4-to-8-year time frame (sHR = 1.33, *p* = 0.39). Age 55 years or older was associated with reduced risk of any invasive subsequent event (sHR = 0.62, *p* = 0.04). ER-PR- disease was also associated with increased risk of subsequent ipsilateral invasive events in the 0-to-4-year time frame (sHR = 2.64, *p* = 0.02) and unchanged risk in the 4-to-8-year time frame (sHR = 1.41, *p* = 0.46). Age 55 years or greater was associated with reduced risk (sHR = 0.44, *p* = 0.006). ER-PR- disease was associated with non-significantly increased risk of contralateral invasive events in the 0-to-4-year time frame (sHR = 2.10, *p* = 0.24) and unchanged risk in the 4-to-8-year time frame (sHR = 1.33, *p* = 0.55). In a time-invariant model, ER-PR- disease was associated with non-significantly increased risk of breast cancer mortality (sHR = 2.36, *p* = 0.16).

### 3.3. Unmatched Breast-Conserving Surgery with Radiation Therapy Cohort

In total, 6925 patients met inclusion criteria for the breast-conserving surgery without radiation cohort and were eligible for matching, among whom 4797 (76.2%) had ER+PR+ disease and 1498 (23.8%) had ER-PR- disease. Median age was 58 years (IQR 50–65) and median lesion size was 9 mm (IQR 5–15). White (77.1%) were white, 607 (9.6%) were Black, and 833 (13.2%) were of a non-white, non-Black race. In total, 666 (10.6%) of patients had low-grade disease, 2639 (41.9%) had intermediate-grade disease, 2353 (37.4%) had high-grade disease, and 637 (10.1%) had undifferentiated-grade disease. During the 8-year follow-up period, 124 (2.0%) patients had an invasive ipsilateral event, 177 (2.8%) had a contralateral invasive event, 33 (0.5%) died of breast cancer, and 240 (3.8%) died of causes other than breast cancer. Within this unmatched cohort, the 8-year cumulative incidence for patients with ER+PR+ relative to ER-PR- lesions was 5.0% vs. 5.6% for any invasive event, 1.7% vs. 2.9% for ipsilateral invasive events, 3.0% vs. 2.5% for contralateral invasive events, and 0.5% vs. 0.7% for breast cancer mortality.

### 3.4. Matched Breast-Conserving Surgery with Radiation Therapy Cohort

A total of 2966 patients were matched at a 1:1 ratio including 1498 patients (50%) with ER-PR- disease and 1498 patients (50%) with ER+PR+ disease (Table 3). Median age was 59 years (IQR 53–66). Median size was 12 mm (IQR 6–17). In total, 2361 (78.8%) of patients were white, 273 (9.1%) of patients were Black, and 362 (12.1%) of patients were of a non-white, non-Black race. Overall 42 (1.4%) patients had low-grade disease, 450 (15.0%) had intermediate-grade disease, 1933 (64.5%) had high-grade disease, and 571 (19.1%) had undifferentiated-grade disease. During the 8-year follow-up period, 71 (2.4%) patients had an ipsilateral invasive event, 85 (2.8%) had a contralateral invasive event, 20 (0.7%) patients died of breast cancer, and 131 (4.4%) patients died of causes other than breast cancer.

**Figure 2:**
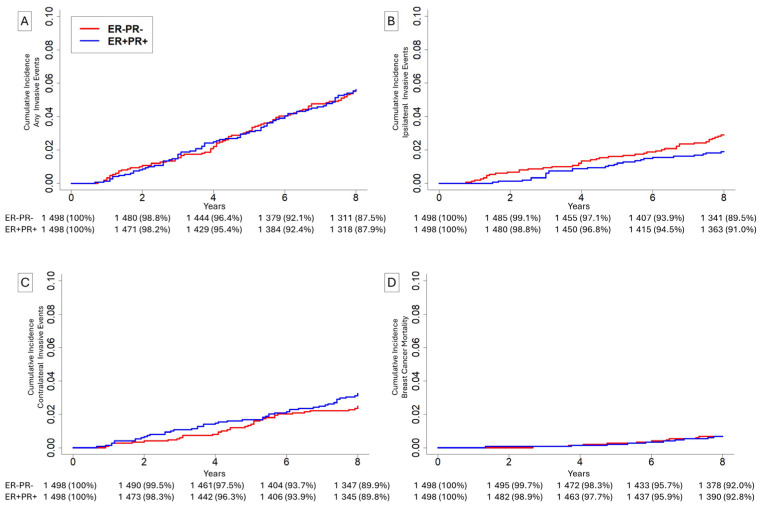
Cumulative incidence of (**A**) any invasive events, (**B**) ipsilateral invasive events, (**C**) contralateral invasive events, and (**D**) breast cancer mortality for patients undergoing breast-conserving surgery with adjuvant radiation with estrogen-receptor-positive (ER+) and progesterone-receptor-positive (PR+) DCIS, or ER-PR- DCIS.

Within this matched cohort, the 8-year cumulative incidence for patients with ER+PR+ relative to ER-PR- lesions was 5.6% vs. 5.6% (Figure 2A, *p* = 0.97) for any invasive event, 1.9% vs. 2.9% (Figure 2B, *p* = 0.07) for ipsilateral invasive events, 3.2% vs. 2.5% (Figure 2C, *p* = 0.22) for contralateral invasive events, and 0.7% vs. 0.7% (Figure 2D, *p* = 0.99) for breast cancer mortality. In adjusted multivariate models (Table 4), ER-PR- disease was associated with unchanged risk of any invasive subsequent event in both the 0-to-4-year time frame (sHR = 0.86, *p* = 0.53) and in the 4-to-8-year time frame (sHR = 1.10, *p* = 0.63). ER-PR- disease was associated with non-significantly increased risk of subsequent ipsilateral invasive events in the 0-to-4-year time frame (sHR = 1.41, *p* = 0.34) and in the 4-to-8-year time frame (sHR = 1.70, *p* = 0.11). ER-PR- disease was associated with non-significantly reduced risk of contralateral invasive events in the 0-to-4-year time frame (sHR = 0.56, *p* = 0.11) and unchanged risk in the 4-to-8-year time frame (sHR = 0.91, *p* = 0.73). In a time-invariant model, ER-PR- disease was associated with unchanged risk of breast cancer mortality (sHR = 1.02, *p* = 0.97).

### 3.5. Matched ER+PR- Breast-Conserving Surgery Without Radiation Therapy Cohort

A cohort of 888 patients was assembled by matching 222 (25%) patients with ER+PR- disease to 222 (25%) patients with ER-PR- disease in a 1:1 fashion and to 444 (50%) patients with ER+PR+ disease in a 1:2 fashion. Median age was 60 years (IQR 53–68). Median size was 10 mm (IQR 5–15). In total, 712 (80.2%) of patients were white, 66 (7.4%) of patients were Black, and 110 (12.4%) of patients were of a non-white, non-Black race. Overall, 25 (2.8%) of patients had low-grade disease, 279 (31.4%) had intermediate-grade disease, 460 (51.8%) had high-grade disease, 124 (14.0%) had undifferentiated-grade disease. During the 8-year follow-up period, 39 (4.4%) patients had an ipsilateral invasive event, 13 (1.5%) had a contralateral invasive event, 9 (1.0%) patients died of breast cancer, and 57 (6.4%) patients died of causes other than breast cancer.

**Figure 3:**
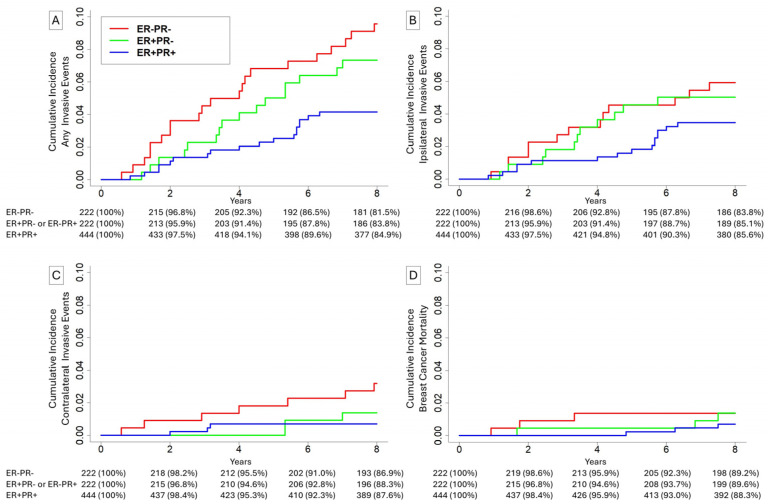
Cumulative incidence of (**A**) any invasive events, (**B**) ipsilateral invasive events, (**C**) contralateral invasive events, and (**D**) breast cancer mortality for patients undergoing breast-conserving surgery without radiation with estrogen-receptor-positive (ER+) and progesterone-receptor-positive (PR+) DCIS, or ER-PR- DCIS, or ER+PR- DCIS.

Within this matched cohort, the 8-year cumulative incidences for patients with ER-PR- lesions, ER+PR- lesions, and ER+PR+ lesions were 9.6%, 7.3%, and 4.1% (Figure 3A, *p* = 0.02), respectively, for any invasive event. The cumulative incidences of ipsilateral invasive events were 5.9%, 5.0%, and 3.5% (Figure 3B, *p* = 0.31), respectively. The cumulative incidences of contralateral invasive events were 3.2%, 0.9%, and 0.7% (Figure 3C, *p* = 0.04), respectively. When patients with ER+PR+ and ER+PR- disease were grouped together, the cumulative incidences of contralateral invasive events for patients with ER+ lesions and ER- lesions were 3.2% and 0.9%, respectively (Figure 3D, *p* = 0.02).

### 3.6. Matched ER+PR- Breast-Conserving Surgery with Radiation Therapy Cohort

A cohort of 2040 patients was assembled by matching 406 (25%) patients with ER+PR- disease to 406 (25%) patients with ER-PR- disease in a 1:1 fashion and to 812 (50%) patients with ER+PR+ disease in a 1:2 fashion. Median age was 59 years (IQR 52–65). Median size was 11 mm (IQR 5–18). In total, 1288 (79.3%) of patients were white, 167 (10.3%) of patients were Black, and 169 (10.4%) of patients were of a non-white, non-Black race. Overall, 1305 (80.4%) had high-grade disease, 319 (19.6%) had undifferentiated-grade disease, and 0 (0%) had low- or intermediate-grade disease. During the 8-year follow-up period, 39 (2.4%) patients had an ipsilateral invasive event, 54 (3.3%) had a contralateral invasive event, 15 (0.9%) patients died of breast cancer, and 64 (3.9%) patients died of causes other than breast cancer.

**Figure 4:**
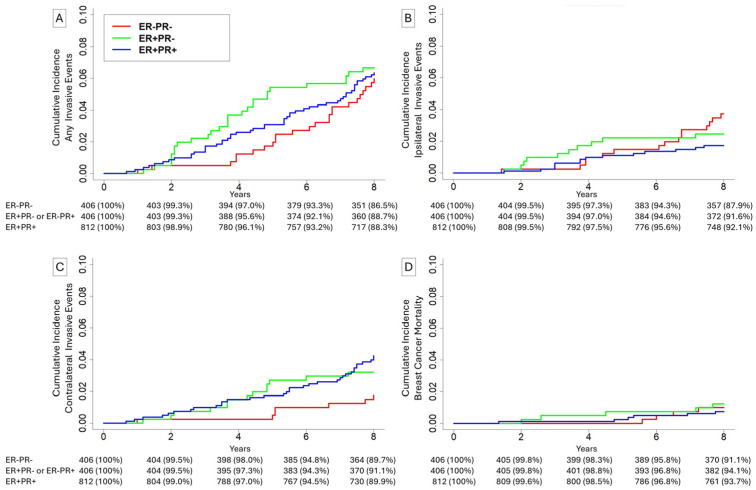
Cumulative incidence of (**A**) any invasive events, (**B**) ipsilateral invasive events, (**C**) contralateral invasive events, and (**D**) breast cancer mortality for patients undergoing breast-conserving surgery with adjuvant radiation with estrogen-receptor-positive (ER+) and progesterone-receptor-positive (PR+) DCIS, or ER-PR- DCIS, or ER+PR- DCIS.

Within this matched cohort, the 8-year cumulative incidences for patients with ER-PR- lesions, ER+PR- lesions, and ER-PR- lesions were 6.0%, 6.7%, and 6.3% (Figure 4A, *p* = 0.89), respectively, for any invasive event. The cumulative incidences of ipsilateral invasive events were 1.7%, 2.5%, and 3.7% (Figure 4B, *p* = 0.11), respectively. The cumulative incidences of contralateral invasive events were 1.7%, 3.2%, and 4.2% (Figure 4C, *p* = 0.07), respectively. When patients with ER+PR- were combined with patients with ER+PR+ disease for analysis, this resulted in a significant difference between groups (Figure 4D, 1.7% vs. 3.9%, *p* = 0.04).

## 4. Discussion

Here we demonstrate in a cohort of patients with DCIS who did not receive endocrine therapy with similar clinicopathologic risk features that patients with ER+PR+ disease have a significantly different risk of ipsilateral and contralateral invasive events after breast-conserving surgery only vs. breast-conserving surgery with radiation therapy relative to patients with ER-PR- disease. Specifically, patients with ER-PR- disease are at increased risk of early invasive ipsilateral recurrence relative to those with ER+PR+ disease when treated with breast-conserving surgery alone, but unchanged risk after breast-conserving surgery with adjuvant external beam radiation. Interestingly, when radiation was administered, patients with ER+PR+ disease appeared to be at a non-significantly increased risk of contralateral subsequent invasive events. Patients with mixed ER and PR status behaved more similarly to ER-PR- disease in the absence of radiation but more similarly to ER+PR+ disease in the presence of adjuvant radiation. Critically, despite the fact that ER and PR are routinely tested in patients with DCIS, neither is incorporated into any of the common clinicopathologic risk scoring systems including the Memorial Sloan Kettering Cancer Center DCIS nomogram [36], the RTOG 9804 trial criteria [12], the ECOG-ACRIN 5194 trial criteria [14], and the Van Nuys Prognostic Index [16].

This finding is significant because one of the primary arguments in support of molecular tests is that they provide information beyond that of traditional clinicopathologic risk factors. Both Oncotype DX DCIS and DCISionRT, two of the most common molecular risk stratification systems, require testing beyond what is performed as part of routine care. Specifically, Oncotype DX DCIS involves the use of RT-qPCR and DCISionRT requires the performance of immunohistochemistry tests beyond standard ER and PR testing. Furthermore, publications regarding the development of both of these tests reported employing non-linear techniques [17,37], which were not used by any of the commonly known purely clinicopathologic risk scoring systems. Taken altogether, these findings suggest that the incorporation of ER and PR status to clinicopathologic models as well as the use of non-linear models may allow for marked improvement in their efficacy.

While efficacy is important for any DCIS risk stratification system, given the overall excellent outcomes for patients with DCIS, it is not just the efficacy but also the value of each scoring system with respect to its cost that should be considered. Notably, in a recent comparison of DCISionRT to traditional clinicopathologic scoring systems, DCISionRT classified 63% of patients in the cohort as high risk and only 37% as low risk. A 2020 cost analysis of DCISionRT found that the test would be cost-effective relative to an approach of irradiating all women with DCIS if the test were priced at $4588, with 10 year ipsilateral invasive rates of 6.3% for the DCISionRT approach and 5.8% for the approach of irradiating all women [38]. An approach of no irradiation was the most cost-effective of the three, with a 10-year ipsilateral invasive rate of 9.1%. A free, publicly available non-linear clinicopathologic risk scoring system incorporating ER and PR status and designed to classify most patients as low risk would represent a more cost-effective approach than DCISionRT and would likely allow for a reduction in the ipsilateral invasive rate beyond the no-irradiation approach.

Though not statistically significant, we also found an interesting trend with respect to the risk of contralateral invasive events. Namely, patients with ER+PR+ disease appeared to have a reduced risk of contralateral invasive events when the treatment approach involved only breast-conserving surgery, but an increased risk when the treatment approach included both breast-conserving surgery and radiation therapy. One possible mechanism for this curious observation could be an abscopal effect from radiation specific to patients with hormone-receptor-negative lesions that may present more immunogenic neoantigens than hormone-receptor-positive lesions. However, given that the majority of DCIS lesions are hormone-receptor-positive, and two of the early DCIS radiotherapy trials found an increased risk of contralateral cancers among patients receiving radiotherapy [39,40], it is perhaps more likely that radiation may increase the risk of contralateral cancers in patients with hormone-receptor-positive DCIS by means such as radiation scatter.

A third finding of our study relates to the risk of subsequent invasive events for patients with ER+PR- lesions. Interestingly, it appeared that these patients had lesions that behaved similarly to ER-PR- lesions with respect to subsequent ipsilateral invasive events, but more similarly to ER+PR+ lesions with respect to contralateral invasive events. Specifically, these patients appeared to be at elevated risk of ipsilateral invasive subsequent events when treated without radiation and elevated risk of contralateral invasive subsequent events when treated with radiation. Given that invasive events, regardless of laterality, are associated with increased risk of ultimate breast cancer mortality for patients with DCIS, the ability to identify differential risks between patients for not only ipsilateral invasive but also contralateral invasive events would be highly useful, particularly given that endocrine therapy administration can be used to modulate that risk [10]. A test that could predict risk for both ipsilateral and contralateral invasive events would be highly useful. Notably, our findings with respect to ER+PR- disease and ipsilateral invasive risk are consistent with the assumptions of Oncotype DX DCIS and DCISionRT, both of which incorporate only PR status into their scoring system—Oncotype DX DCIS reported that PR status appeared to be predictive of invasive progression risk whereas ER status appeared to be more predictive of response to endocrine therapy. Our finding that patients with ER+PR- disease had a more similar contralateral invasive risk to patients with ER+PR+ disease, however, is not something for which we are aware of prior literature assessing.

There are several strengths of this study. We used information gleaned from prior studies in the construction of the cohorts. For example, we only included patients who underwent external beam radiation therapy, as those who receive brachytherapy are at increased risk for invasive subsequent events [41], and we excluded patients with very large DCIS to avoid confounding because of its association with diffuse histopathological growth distribution [42]. There are important limitations to our study as well. As a retrospective study, we could not account for unobserved variables that may have resulted in confounding. Several known confounders could not be accounted for as they are not offered by SEER, including margin status, histopathological growth distribution, family history, and whether lesions were diagnosed by screening or an exam. HER2 status, for which the prognostic relevance in DCIS has been debated, was also not included in this study and was not available for the overwhelming majority of patients with DCIS in SEER in the time frame of the study.

## 5. Conclusions

Here we demonstrate that ER and PR status carry independent prognostic and therapeutic implications beyond those of traditional clinicopathologic risk factors like tumor grade and size. Patients with ER+PR- lesions appear to have a subsequent ipsilateral invasive risk comparable to that of patients with ER-PR- lesions but a contralateral invasive risk similar to that of patients with ER+PR+ lesions. A non-linear clinicopathologic model incorporating ER and PR status may allow for the prediction of both ipsilateral and contralateral invasive risk and may offer greater predictive value than traditional purely clinicopathologic linear models and be more cost-effective than molecular tests like DCISionRT and Oncotype DX DCIS. Further work is needed to explore the viability of such a model.

## Figures and Tables

**Table 1:** Characterization of matched patients undergoing breast-conserving surgery without adjuvant radiation for ER+PR+ and ER-PR- DCIS, with 2:1 matching.

	ER+PR+ DCIS (*n* = 738)	ER-PR- DCIS (*n* = 369)	All Patients (*n* = 1107)
Age			
Median (IQR)	59 (52–66)	60 (52–67)	59 (52–67)
Race			
White	580 (78.6%)	289 (78.3%)	869 (78.5%)
Black	60 (8.1%)	37 (10%)	97 (8.8%)
Other	98 (13.3%)	43 (11.7%)	141 (12.7%)
Grade			
Low	14 (1.9%)	7 (1.9%)	21 (1.9%)
Intermediate	138 (18.7%)	69 (18.7%)	207 (18.7%)
High	457 (61.9%)	227 (61.5%)	684 (61.8%)
Undifferentiated	129 (17.5%)	66 (17.9%)	195 (17.6%)
Size			
Median (IQR)	10 (5–16)	10 (5–18)	10 (5–17)
Year Diagnosis			
2007–2009	400 (54.2%)	218 (59.1%)	618 (55.8%)
2010–2011	338 (45.8%)	151 (40.9%)	489 (44.2%)
Adjuvant Radiation			
Received	0 (0%)	0 (0%)	0 (0%)
Not received	738 (100%)	369 (100%)	1107 (100%)
Adjuvant Endocrine			
Received	0 (0%)	0 (0%)	0 (0%)
Not received	738 (100%)	369 (100%)	1107 (100%)

IQR = interquartile range, ER = estrogen receptor, PR = progesterone receptor.

**Table 2:** Competing risks regressions for matched cohort of DCIS patients undergoing breast-conserving surgery without adjuvant radiation for ER+PR+ or ER-PR- DCIS.

	Any Invasive Event		Ipsilateral Invasive Event		Contralateral Invasive Event	
	sHR (95% CI)	*p*	sHR (95% CI)	*p*	sHR (95% CI)	*p*
Age						
≥55 years	Ref	-	Ref	-	Ref	-
<55 years	1.61 (1.03–2.53)	0.038	2.28 (1.27–4.09)	0.006	0.93 (0.42–2.06)	0.85
Race						
Non-Black	Ref	-	Ref	-	Ref	-
Black	1.16 (0.56–2.38)	0.69	1.50 (0.64–3.52)	0.35	0.37 (0.05–2.76)	0.34
Grade						
Low or Intermediate	Ref	-	Ref	-	Ref	-
High or Undifferentiated	1.15 (0.65–2.04)	0.62	1.20 (0.57–2.51)	0.64	1.19 (0.45–3.14)	0.73
Size						
<15 mm	Ref	-	Ref	-	Ref	-
10–30 mm	0.73 (0.45–1.18)	0.19	0.82 (0.45–1.51)	0.53	0.47 (0.19–1.16)	0.10
ERPR Status						
ER+PR+	Ref	-	Ref	-	Ref	-
ER-PR- 0–4 years	2.47 (1.29–4.72)	0.007	2.64 (1.19–5.86)	0.017	2.10 (0.61–7.29)	0.24
ER-PR- 4–8 years	1.33 (0.70–2.53)	0.39	1.41 (0.57–3.47)	0.46	1.33 (0.52–3.40)	0.55

sHR = subdistribution hazard ratio, CI = confidence interval, BCS = breast-conserving surgery, RT = radiation therapy, ER = estrogen receptor, PR = progesterone receptor.

**Table 3:** Characterization of matched patients undergoing breast-conserving surgery with adjuvant radiation for ER+PR+ and ER-PR- DCIS, with 1:1 matching.

	ER+PR+ DCIS (*n* = 1498)	ER-PR- DCIS (*n* = 1498)	All Patients (*n* = 2996)
Age			
Median (IQR)	59 (53–66)	60 (53–66)	59 (53–66)
Race			
White	1200 (80.1%)	1161 (77.5%)	2361 (78.8%)
Black	138 (9.2%)	135 (9%)	273 (9.1%)
Other	160 (10.7%)	202 (13.5%)	362 (12.1%)
Grade			
Low	21 (1.4%)	21 (1.4%)	42 (1.4%)
Intermediate	225 (15%)	225 (15%)	450 (15%)
High	973 (65%)	960 (64.1%)	1933 (64.5%)
Undifferentiated	279 (18.6%)	292 (19.5%)	571 (19.1%)
Size			
Median (IQR)	11 (6–17)	12 (6–18)	12 (6–17)
Year Diagnosis			
2007–2010	845 (56.4%)	872 (58.2%)	1717 (57.3%)
2011–2013	653 (43.6%)	626 (41.8%)	1279 (42.7%)
Adjuvant Radiation			
Received	1498 (100%)	1498 (100%)	2996 (100%)
Not received	0 (0%)	0 (0%)	0 (0%)
Adjuvant Endocrine			
Received	0 (0%)	0 (0%)	0 (0%)
Not received	1498 (100%)	1498 (100%)	2996 (100%)

IQR = interquartile range, ER = estrogen receptor, PR = progesterone receptor.

**Table 4:** Competing risks regressions for matched cohort of DCIS patients undergoing breast-conserving surgery with adjuvant radiation for ER+PR+ or ER-PR- DCIS.

	Any Invasive Event		Ipsilateral Invasive Event		Contralateral Invasive Event	
	sHR (95% CI)	*p*	sHR (95% CI)	*p*	sHR (95% CI)	*p*
Age						
≥55 years	Ref	-	Ref	-	Ref	-
<55 years	1.18 (0.86–1.63)	0.31	2.12 (1.33–3.38)	0.0016	0.73 (0.45–1.20)	0.21
Race						
Non-Black	Ref	-	Ref	-	Ref	-
Black	1.48 (0.93–2.34)	0.096	1.65 (0.84–3.24)	0.14	1.05 (0.51–2.19)	0.89
Grade						
Low or Intermediate	Ref	-	Ref	-	Ref	-
High or Undifferentiated	1.17 (0.76–1.81)	0.47	1.39 (0.69–2.81)	0.35	0.98 (0.55–1.74)	0.95
Size						
<15 mm	Ref	-	Ref	-	Ref	-
10–30 mm	1.27 (0.93–1.73)	0.13	1.25 (0.78–2.00)	0.36	1.24 (0.81–1.90)	0.33
ERPR Status						
ER+PR+	Ref	-	Ref	-	Ref	-
ER-PR- 0–4 years	0.86 (0.53–1.39)	0.53	1.41 (0.69–2.89)	0.34	0.56 (0.28–1.14)	0.11
ER-PR- 4–8 years	1.10 (0.74–1.63)	0.63	1.70 (0.89–3.22)	0.11	0.91 (0.53–1.56)	0.73

sHR = subdistribution hazard ratio, CI = confidence interval, BCS = breast-conserving surgery, RT = radiation therapy, ER = estrogen receptor, PR = progesterone receptor.

## Data Availability

The data underlying this article were provided by NCI/SEER by permission. Data can be requested through NCI/SEER.
